# The Influence of Processing Speed, Attention, and Inhibition on Texas Functional Living Scale Performance

**DOI:** 10.1093/arclin/acac029

**Published:** 2022-05-22

**Authors:** Scott Roye, John F Linck, Jordan Hoffmeister, Christopher T Copeland

**Affiliations:** Neuropsychology Service, Department of Psychiatry and Behavioral Sciences, University of Oklahoma Health Sciences Center, Oklahoma City, OK, USA; Neuropsychology Service, Department of Psychiatry and Behavioral Sciences, University of Oklahoma Health Sciences Center, Oklahoma City, OK, USA; Neuropsychology Service, Department of Psychiatry and Behavioral Sciences, University of Oklahoma Health Sciences Center, Oklahoma City, OK, USA; Neuropsychology Service, Department of Psychiatry and Behavioral Sciences, University of Oklahoma Health Sciences Center, Oklahoma City, OK, USA

**Keywords:** Assessment, Everyday functioning, Attention, Elderly/geriatrics/aging

## Abstract

**Objective:**

Attention, inhibition, and processing speed are related to functional decline among older adults. This study attempts to clarify the relationships between these cognitive factors and adaptive functioning.

**Method:**

We examined relationships between attention, inhibition, and processing speed, with scores on the Texas Functional Living Scale (TFLS), a performance-based measure of daily functioning, in a mixed clinical sample of 530 older adults who were referred for an outpatient neuropsychological evaluation.

**Results:**

The current study used a confirmatory factor analysis (CFA) to derive a three-factor cognitive model consisting of attention, inhibition, and processing speed. Results from a hierarchical regression, which included factor scores from the CFA, revealed that processing speed was the only significant predictor of TFLS performance when all three cognitive factors were included within a single model.

**Conclusion:**

These results highlight the influence of processing speed as an important indicator of functional decline among a clinical population of older adults.

## Introduction

Cognitive and functional decline can limit autonomy in older adulthood, particularly among those with neurocognitive disorders. Therefore, early identification of cognitive and functional decline in aging populations is important in the diagnosis and management of neurodegenerative conditions. Although the negative relationship between age and cognition has been well established (Nickl-Jockschat et al., 2012; Salthouse, 2003, 2010), with changes often manifesting as declines in functional capabilities (Gold, 2012; Nikolova, Demers, & Béland, 2009), it is known that independence for activities of daily living (ADLs) or instrumental activities of daily living (IADLs) are also dependent upon motor and perceptual capabilities (Mlinac & Feng, 2016). Additionally, the impact of environmental processes and coping related factors have been considered when examining contributors to disability status (Barberger-Gateau, Fabrigoule, Amieva, Helmer, & Dartigues, 2002; Verbrugge & Jette, 1994). However, much of the research remains focused on the association between neuropsychological functioning and the ability to manage ADLs/IADLs.

Prior research evaluating the impact of cognition on ADLs and IADLs has broadly supported the role of executive functioning (EF) in evolving impairment in older populations (Back-Madruga et al., 2002; Bell-McGinty, Podell, Franzen, Baird, & Williams, 2002; Boyle et al., 2003; Farias, Harrell, Neumann, & Houtz, 2003; Jefferson, Paul, Ozonoff, & Cohen, 2006), whereas other studies have highlighted the influence of non-EF-related cognitive functions (Hall, Vo, Johnson, Barber, & O’Bryant, 2011; Hinkin et al., 2002). Glisky (2007) proposed that age-related reductions in attention (Craik, 1986), processing speed (Salthouse, 1996), and inhibition (Hasher, Zacks, & May, 1999) may influence ADL/IADL functioning, with research identifying specific links between reduced attention and processing speed and decreased mobility (Owsley & McGwin Jr., 2004) and worse driving (Anstey, Windsor, Luszcz, & Andrews, 2006; Edwards et al., 2006; Edwards, Bart, O’Connor, & Cissell, 2010).

In fact, prior neuroimaging studies generally support this theory in showing reduced white matter integrity and reduced gray matter volume in areas commonly associated with cognitive slowing, attention, and disinhibition in advancing age (Brugulat-Serrat et al., 2020; Burgmans et al., 2011; Hedden & Gabrieli, 2004; Hong et al., 2015; Ishikawa, Meguro, Ishii, Tanaka, & Yamaguchi, 2012; Kerchner et al., 2012; MacPherson et al., 2017; Turken et al., 2008; Wolf et al., 2014).

The role of processing speed appears particularly relevant, as neuropsychological measures designed to assess processing speed have demonstrated prognostic value in differentiating and monitoring the progression of neurocognitive disorders (Lu, Chan, Fung, & Lam, 2016; Lu, Chan, & Lam, 2017; Mortamais et al., 2017) as well as serving to detect subtle functional changes among individuals with Alzheimer’s disease (Marson, 2015). Research further suggests that general cognitive slowing is associated with aging and may confound multiple cognitive processes (Salthouse, 1996), including general intelligence (Betjemann et al., 2010), attention (Lustig, Hasher, & Tonev, 2006; Stawski, Sliwinski, & Hofer, 2013), and EF (Albinet, Boucard, Bouquet, & Audiffren, 2012; Rey-Mermet & Gade, 2018; Salthouse, 2005). More recent studies suggest that the structure of an objective-testing measure, particularly those containing a speeded component, can potentially confound performance results. For example, a meta-analysis by Rey-Mermet and Gade (2018) examining inhibition and aging suggests that inconsistent findings may be related to task selection or how inhibition is considered, and the authors recommend examining speed-accuracy trade-offs in order to further investigate this relationship. Consistent with this idea, Lassen-Greene et al. (2017) demonstrated that individuals with mild cognitive impairment (MCI) were slower, but similarly accurate when compared to those without MCI. Moreover, studies have suggested that accounting for processing speed statistically may improve the specificity of test performance interpretations (Albinet et al., 2012; Karr, Hofer, Iverson, & Garcia-Barrera, 2019; Nigg et al., 2017; Roye et al., 2020).

The Texas Functional Living Scale (TFLS; Cullum, Weiner, & Saine, 2009) is an objective-performance measure designed to assess adaptive functioning among individuals who are 16–90 years of age. The TFLS contains 24 items intended to measure time and money management, communication skills, and memory. Although TFLS performance is assessed across five individual scales, including an additive Total score, factor analytic studies demonstrate support for the clinical utility of the Total score performance, as this was the only scale to consistently demonstrate adequate convergent and discriminant validity with other neurocognitive measures (Gonzalez, Soble, Marceaux, & McCoy, 2017; Lowe, Nguyen, Copeland, & Linck, 2020). Earlier versions of the TFLS have demonstrated good sensitivity when measuring functional change among those diagnosed with Alzheimer’s disease (Weiner, Fields, Hynan, & Cullum, 2008) and have demonstrated good discriminant validity when determining level of care among individuals diagnosed with dementia (Weiner et al., 2007). Regarding the measure’s relationship with individual cognitive domains, Total score performance on the TFLS has demonstrated moderate correlations with both abstract reasoning and processing speed indices within a large, non-clinical adult sample (Drozdick & Cullum, 2011).

Other studies examining TFLS performance in veteran and non-veteran mixed outpatient samples similarly found moderate to large correlations between the TFLS and both EF and processing speed measures as well as other measures of intelligence (Gonzalez et al., 2017; Nguyen et al., 2019). Unique to previous studies, results from Nguyen and colleagues (2019) demonstrated that a measure of shifting and abstract reasoning significantly predicted TFLS performance after controlling for non-EF performances (i.e., memory, visuospatial ability, processing speed, language, and attention). Notably, speeded measures were included within both EF and non-EF domains, within domain relationships were derived theoretically instead of statistically, and, although multiple measures were used to determine EF and non-EF performance, individual cognitive domains within these two constructs were not always assessed using multiple measures (i.e., processing speed). Therefore, this suggests that further examination of relationships between the TFLS and domain-specific cognitive performance may be warranted.

The purpose of the current study is to examine the relationships between attention, inhibition, processing speed, and objective IADL performance, as measured by the TFLS. Although previous studies have explored relationships between TFLS performance and cognition (Drozdick & Cullum, 2011; Gonzalez et al., 2017; Nguyen et al., 2019), to our knowledge, the predictive influence of attention, inhibition, and processing speed have never been directly compared to this measure. This study attempts to clarify previously identified relationships between cognition and adaptive functioning by comparing statistically driven latent cognitive factors comprised of multiple performance measures and their predictive influence on TFLS performance.

## Materials and Methods

### Participants and Procedures

Participants included in this study were adult patients referred for a neuropsychological evaluation at an academic medical outpatient clinic between January 2016 and June 2021. The process of obtaining archival data via electronic medical records was approved by the local Institutional Review Board. Assessments were conducted by licensed clinical neuropsychologists, psychometricians, and pre-doctoral and post-doctoral psychology trainees, who were supervised by a licensed neuropsychologist. Assessments were conducted using a flexible battery approach. Missing data were managed using a pairwise deletion method for correlation and hierarchical regression analyses, whereas multiple imputation was used for the confirmatory factor analysis (CFA). A patient’s data were included if the individual completed a TFLS during their assessment, was at least 60 years old, and provided valid effort, as indicated by their performance on standalone and/or embedded validity measures (see in the following). Additionally, performance variables were standardized within the sample and outliers (*z*-scores = ±3.5) were removed prior to analyses. Frequency totals of each variable can be found in Table 1. Overall, 11 individuals were excluded from analyses and the final sample consisted of 530 individuals as part of a mixed clinical sample. Participants were between 60 and 94 years old (*M* = 74.7 years; standard deviation [*SD*] = 7.4) and had an average education of 13.8 years (*SD* = 3.0). Most of the sample identified as White (89.4%), and just over half identified as women (55.1%). Further demographic and diagnostic characteristics of the sample are described in Table 1.

**Table 1:** Demographic information

Characteristics		*n*	All patients	No diagnosis	MCI/mild NCD	Major NCD
*N*		—	530	215	136	179
Age		—	74.58 (7.35)	73.9 (7.64)	73.60 (6.74)	76.13 (7.25)
Education		—	13.89 (2.67)	13.89 (2.59)	13.94 (2.75)	13.84 (2.77)
Gender (women)		—	56% (*n* = 297)	58% (*n* = 125)	56% (*n* = 71)	61% (*n* = 101)
Race/ethnicity (%)						
Caucasian		—	89.20%	91.20%	88.80%	89.80%
African American		—	6.20%	5.60%	6.70%	6.80%
Latinx		—	1.50%	0.90%	2.20%	1.70%
Other		—	0.90%	0.90%	2.20%	1.70%
Cognitive performance						
CPT omission		268	14.11 (19.67)	13.91 (20.72)	9.96 (13.77)	19.93 (22.71)
CPT ß		268	1.49 (1.98)	1.47 (1.91)	1.59 (2.49)	1.41 (1.27)
RBANS digit span		505	8.77 (2.26)	8.90 (2.42)	9.02 (2.25)	8.43 (2.02)
RBANS coding		505	27.40 (11.56)	29.64 (11.82)	30.44 (10.16)	21.92 (10.79)
SCWT word		204	51.31 (15.74)	76.15 (21.30)	83.11 (18.38)	70.32 (16.03)
SCWT color		204	76.74 (19.60)	51.67 (16.18)	57.14 (15.12)	43.94 (12.74)
^a^SCWT interference		204	−0.01 (1.00)	0.03 (0.91)	0.14 (1.17)	−0.22 (0.90)
TMT A (s)		513	64.44 (43.95)	57.00 (35.95)	55.34 (33.93)	80.88 (54.41)
TFLS total		530	36.15 (10.16)	38.07 (9.33)	40.32 (6.96)	30.70 (10.86)

*Note.* MCI = mild cognitive impairment; NCD = neurocognitive disorder; CPT = Conners’ Continuous Performance Test II; RBANS = Repeatable Battery for the Assessment of Neuropsychological Status; SCWT = Stroop Color Word Test; TMT = Trail Making Test; TFLS = Texas Functional Living Scale.

^a^Standardized residual score.

### Measures

#### Performance validity

Objective performance validity was determined based on a patient’s performance on a combination of the following measures: Test of Memory Malingering (Tombaugh, 1996), Wechsler Adult Intelligence Scale-fourth edition (WAIS-IV; Wechsler, 2008), digit span, reliable digit span, and California Verbal Learning Test-second edition ( Delis, Kaplan, Kramer, & Ober, 2000), forced choice. Validity scores were set in accordance with standard, manual-based cut-off scores.

#### Attention

Attention was assessed using omission errors from the Conners’ Continuous Performance Test II (CPT-II; Conners et al., 2004), a computerized test of sustained attention and response inhibition. Omission errors, or the frequency of nontargets a subject failed to respond to, are commonly used as a measure of attention (Delisle & Braun, 2011; Homack & Riccio, 2006; Malloy-Diniz, Fuentes, Leite, Correa, & Bechara, 2007; Strauss, Sherman, & Spreen, 2006) and have high internal consistency and test–retest reliability (Conners et al., 2004; Strauss et al., 2006). A second measure of attention came from the Digit Span subtest of the Repeatable Battery for the Assessment of Neuropsychological Status (RBANS; Randolph, 2012). Digit Span requires participants to verbally repeat sequences of numbers in the same order as the examiner and has demonstrated moderate to strong convergent validity with the WAIS-IV Digit Span subtest in clinical and non-clinical samples (Calamia, Roye, & Lemke, 2018; McKay et al., 2007).

#### Inhibition

The first measure of inhibition was response style (β) from the CPT-II. This variable is expressed as a function of speed/accuracy trade-off and is used to detect impulsivity. Response style has demonstrated fair test–retest reliability independently and good test–retest reliability when combined with CPT-II indices for neurological assessment (.92; Homack & Riccio, 2006). Second, the Color-Word Interference trial from the Stroop Color Word Test (SCWT; Golden, 1978) was included as a measure of inhibition. The SCWT has been well established as a measure of inhibition (Lucas et al., 2005; Steinberg, Bieliauskas, Smith, & Ivnik, 2005; Verhaeghen & De Meersman, 1998) and demonstrates diagnostic sensitivity among older adults with dementia (Amieva, Phillips, Della Sala, & Henry, 2004; Ben-David, Tewari, Shakuf, & Van Lieshout, 2014). As it has been suggested that the processing speed component of this task may affect performance among older adults with dementia (Ben-David et al., 2014), a standardized residual score was created by regressing out performance on the first two trials from the Interference trial (Nigg et al., 2017).

#### Processing speed

The processing speed factor consisted of four speeded variables, two tasks requiring oral responses and two requiring a motor component. Speeded measures requiring oral responses included the Word and Color trials from the SCWT. Motor tasks included the Coding subtest from the RBANS and Trails A from the Trail Making Test (TMT; Reitan, 1958). Although Coding is part of the Attention Index on the RBANS, the task emulates the Coding subtest from the WAIS-IV, which was designed to measure processing speed. Both tasks demonstrate strong convergent validity with one another (*r* = .83; McKay et al., 2007). Additionally, TMT-A is recognized as a measure of output speed (Nigg et al., 2005, 2017; Strauss et al., 2006).

#### Texas Functional Living Scale

The TFLS is a 24-item performance-based measure designed to assess IADLS. It contains five subscales: Time, Money and Calculation, Communication, and Memory, as well as a Total score to interpret an overall level of functioning. *T*-scores can be determined for each subscale with interpretations ranging from “High Average” to “Severely Impaired.” The TFLS exhibits adequate validity and reliability with multiple measures of adaptive functioning (Cullum et al., 2009). Internal consistency within the current sample was .78.

### Data Analyses

**Fig. 1:**
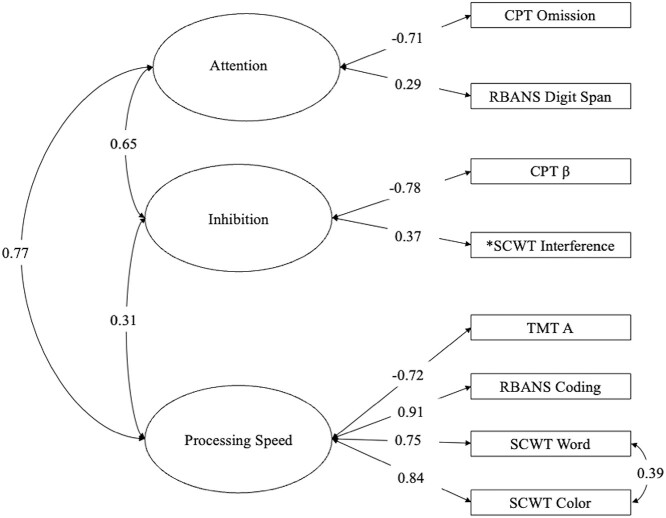
Three-factor models of cognition. CPT = Conners’ Continuous Performance Test; RBANS = Repeatable Battery for the Assessment of Neuropsychological Status; β = CPT Response Style; SCWT = Stroop Color Word Test; TMT = Trail Making Test. ^*^Indicates a value that was residualized of variance attributable to a control variable.

Raw performance scores were used to avoid reducing measure variance with demographically normed performance scores. A CFA was estimated on the cognitive constructs each variable was designed to assess (see Fig. 1). Analyses were conducted using MPlus Version 7 (Muthén & Muthén, 2012) to test the fit of the proposed three-factor model. Multiple fit statistics were interpreted to determine goodness of fit, including Pearson chi-square, Comparative Fit Index (CFI) value >.90 (Bentler, 1990), Root Mean Square Error of Approximation (RMSEA) ≤.08 with a probability RMSEA ≤.05 (Steiger, 1990), and Standardized Root Mean Square Residual (SRMR) <.08 (Brown, 2014). Latent variable factor scores were extracted from the CFA and included within the correlation and multiple regression analyses, which were analyzed using SPSS version 26.

## Results

### Preliminary Analyses

Regarding the hierarchical regression, the assumptions of normality, linearity, and homoscedasticity of residuals were met. Additionally, prior to running a hierarchical linear regression analysis, the data were assessed for multicollinearity. Variance inflation factors were determined to be less than four, which indicated acceptable levels of multicollinearity (O’Brien, 2007). Hierarchical linear regressions were conducted to examine the unique contributions of latent cognitive factors while controlling for potential confounding demographic influences. Notably, the cognitive variables were factor scores extracted from a CFA.

Specifically, Step 1 of the regression included age and education to account for demographic variance, whereas Step 2 included three cognitive variables derived from factor scores: attention, inhibition, and processing speed.

### Confirmatory Factor Analysis

The three-factor model of cognitive performance indicated an inadequate fit (SRMR = .09). The modification indices within the model output noted that a residual correlation between two scores from the same subtest (SCWT Color and Word trials) would improve model fit (M.I. = 11.84), suggesting they may have a relationship beyond what is being calculated by the individual factors. After accounting for shared variance between these two scores, model fit was adequate for the proposed three-factor model (CFI = .94, RMSEA = .07, SRMR =.07). See Fig. 1 for the CFA model.

### Correlations and Hierarchical Linear Regression Model

Correlations between latent cognitive factors, TFLS total score, and demographic variables are presented in Table 2. Results indicated that all three cognitive factors were positively related to TFLS performance, suggesting that better cognitive performance was related to better performance on the TFLS. Regarding the demographic variables, significant, negative relationships were observed between age and all performance-based measures, whereas years of education was positively related to all performance-based measures. Gender was not significantly related to any objective performance measures.

**Table 2:** Relationships between demographic characteristics and cognitive performance

Correlations among potential predictors	1	2	3	4	5
1. TFLS total	—				
2. Age	−.17^*^	—			
3. Education	.12^*^	−.11	—		
4. Attention	.58^*^	.18^*^	.19^*^	—	
5. Inhibition	.33^*^	.11	−.11	.76^*^	—
6. Processing speed	.62^*^	−.18^*^	−.15^*^	.92^*^	.50^*^

*Note:* TFLS = Texas Functional Living Scale. ^*^p < .01.

Results from the hierarchical regression (see Table 3) indicated that both age and years of education significantly predicted TFLS performance (ß = −.168, *p* < .001; ß = .100, *p* = .021) in Step 1. Step 2, which included all three cognitive factors, accounted for approximately 34% of the total variance in predicting TFLS performance. Within this model, only processing speed was predictive of TFLS performance (ß = .409, *p* = .003).

**Table 3:** Hierarchical regression analysis of predictors of TFLS Total score

		Unstandardized coefficients		Standardized coefficients					
Step	Predictor	*B*	SE	ß	*p*	*R* ^2^	*R* ^2^ Change	*F*	*p*
1						.042	.042	11.306	.000
	Age	−.231	.059	−.168	.000				
	Education	.375	.162	.100	.021				
2						.385	.343	64.747	.000
	Age	−.084	.049	−.061	.082				
	Education	.066	.132	.017	.621				
	Attention	3.215	2.351	.252	.172				
	Inhibition	−1.274	1.355	−.080	.348				
	Processing speed	4.482	1.511	.409	.003				

*Note. N* = 530; SE = standard error of B; Attention = factor score of attention measures; Inhibition = factor score of inhibition measures; Processing speed = factor score of processing speed measures.

## Discussion

The purpose of the current study was to examine the utility of attention, processing speed, and inhibition as they related to adaptive functioning performance, which was measured by TFLS Total score. Results indicated that processing speed was the strongest predictor of TFLS Total performance among an older adult clinical sample. Findings further suggested that processing speed accounted for variance beyond that of attention and inhibition, which were not significantly predictive of TFLS Total score. Overall, the current findings support previous research suggesting the importance of processing speed as it relates to functional decline among older adults. Additionally, these findings may support the prioritization of processing speed measures when assessing daily functioning in a clinical setting.

Results are suggestive of an important relationship between processing speed and adaptive functioning. Specifically, analyses indicated that processing speed accounted for approximately 41% of the variance when both attention and inhibition factors were included in the same model. Previous research (Lassen-Greene et al., 2017; Wadley et al., 2020) similarly identified significant differences in speed and objective IADL performance between older adults with MCI and very early Alzheimer’s disease as well as between individuals with and without MCI. Their research further demonstrated larger speed-accuracy disparities between those with and without MCI. Although the TFLS contains a Memory subscale and does not include a speeded scoring component, Drozdick and Cullum (2011) demonstrated the strongest correlations between TFLS performance and the WAIS-IV Processing Speed Index (*r* = .81) within their sample of individuals diagnosed with Alzheimer’s disease while the WMS-IV memory indices correlations ranged from *r* = .57 to *r* = .67. These findings, combined with those from the current study, support the role of cognitive processing speed as an underlying factor for adaptive functioning among an aging sample (Marson, 2015) via its predictive relationship to TFLS Total performance.

Unique to previous studies examining TFLS performance, this is the first study, to our knowledge, to account for processing speed embedded within objective performance measures, and the first to construct a latent variable model of unique measures. Although past studies have compared index scores from the RBANS, WMS-IV, and WAIS-IV (Drozdick & Cullum, 2011; Nguyen et al., 2019), standardized scores found within those indices have the potential to limit the generalizability of performances, as raw scores are absent of normed demographics. Notably, the current study did not make direct comparisons between objective performance measures previously identified as being related to TFLS performance (i.e., Memory and EF). Although inhibition is an EF domain (Miyake et al., 2000), Nguyen and colleagues (2019) identified a measure of set-shifting and verbal abstract reasoning as the best predictors of TFLS performance after accounting for non-EF performances (i.e., processing speed). However, the breadth of EF and non-EF measures included in their analyses should be considered, as this can increase the likelihood of shared variance within and between EF and non-EF measures and may artificially reduce the predictive power of certain variables. This suggests that future studies may benefit from further specifying adequate predictors of IADL performance by using a latent variable approach, removing specific factors that may confound performance (i.e., processing speed) and comparing such constructs to TFLS performance.

This study is not without limitations. First, although providing a flexible battery is common within a clinical setting, it can result in test selection bias and uneven distribution of cognitive measures within a research sample. For example, participants were only included in the current sample if they completed the TFLS during a neuropsychological evaluation. The inclusion of other performance measures was variable. Additionally, patients assumed to be “too impaired” or “functionally intact” may not have been given a TFLS during their evaluation, potentially skewing the current sample. However, the sample’s size and similar clinical presentation to past studies examining the TFLS (Gonzalez et al., 2017; Lowe et al., 2020; Nguyen et al., 2019) suggest adequate generalizability of the current findings. Next, although the cognitive model proposed in the current study is grounded theoretically, the measures were limited to those available, resulting in an uneven number of measures used to construct each latent variable. Although this is a notable concern, each factor included multiple variables for each cognitive domain and the model demonstrated a good fit within the current sample. Lastly, the processing speed factor was constructed using more variables, which may account for a more robust processing speed variable within the three-factor, CFA model. However, this should not affect the predictive variance of the three cognitive factors and their relationship to adaptive functioning performance, as factor scores were standardized prior to being entered into the regression model. Given the current findings and previous research supporting the relationship between processing speed and adaptive functioning, future studies may benefit from examining the influence of processing speed on the relationship between EF and adaptive functioning. Additionally, future studies could also compare TFLS performances with and without an embedded timed component.

Overall, findings from the current study provide support for a relationship between processing speed and adaptive functioning among older adults. Although previous studies have examined the relationship between TFLS performance and other cognitive domains, to our knowledge, no studies have directly examined relationships between attention, inhibition, and processing speed, nor have previous studies accounted for speeded components embedded within neuropsychological measures, when identifying cognitive relationships. Future studies are encouraged to replicate these findings as well as to compare the influence of processing speed to previously identified cognitive relationships with the TFLS.

## Funding

None to disclose.

## Conflict of Interest

None declared.

## References

[ref1] Albinet, C. T., Boucard, G., Bouquet, C. A., & Audiffren, M. (2012). Processing speed and executive functions in cognitive aging: How to disentangle their mutual relationship? Brain and Cognition, 79(1), 1–11. 10.1016/j.bandc.2012.02.001. 22387275

[ref2] Amieva, H., Phillips, L. H., Della Sala, S., & Henry, J. D. (2004). Inhibitory functioning in Alzheimer’s disease. Brain, 127(5), 949–964. 14645147 10.1093/brain/awh045

[ref3] Anstey, K. J., Windsor, T. D., Luszcz, M. A., & Andrews, G. R. (2006). Predicting driving cessation over 5 years in older adults: Psychological well-being and cognitive competence are stronger predictors than physical health. Journal of the American Geriatrics Society, 54(1), 121–126. 16420208 10.1111/j.1532-5415.2005.00471.x

[ref4] Back-Madruga, C., Boone, K. B., Briere, J., Cummings, J., McPherson, S., Fairbanks, L., et al. (2002). Functional ability in executive variant Alzheimer’s disease and typical Alzheimer’s disease. The Clinical Neuropsychologist, 16(3), 331–340. 10.1076/clin.16.3.331.13846. 12607146

[ref5] Barberger-Gateau, P., Fabrigoule, C., Amieva, H., Helmer, C., & Dartigues, J. F. (2002). The disablement process: A conceptual framework for dementia-associate disability. Dementia and Geriatric Cognitive Disorders, 13(2), 60–66. 11844886 10.1159/000048635

[ref6] Bell-McGinty, S., Podell, K., Franzen, M., Baird, A. D., & Williams, M. J. (2002). Standard measures of executive function in predicting instrumental activities of daily living in older adults. International Journal of Geriatric Psychiatry, 17(9), 828–834. 10.1002/gps.646. 12221656

[ref7] Ben-David, B. M., Tewari, A., Shakuf, V., & Van Lieshout, P. H. (2014). Stroop effects in Alzheimer's disease: Selective attention speed of processing, or color-naming? A meta-analysis. Journal of Alzheimer's Disease, 38(4), 923–938. 10.3233/JAD-131244 24100125

[ref8] Bentler, P. M. (1990). Comparative fit indexes in structural models. Psychological Bulletin, 107(2), 238. 2320703 10.1037/0033-2909.107.2.238

[ref9] Betjemann, R. S., Johnson, E. P., Barnard, H., Boada, R., Filley, C. M., Filipek, P. A., et al. (2010). Genetic covariation between brain volumes and IQ, reading performance, and processing speed. Behavior Genetics, 40(2), 135–145. 20072853 10.1007/s10519-009-9328-2 PMC3608477

[ref10] Boyle, P. A., Malloy, P. F., Salloway, S., Cahn-Weiner, D. A., Cohen, R., & Cummings, J. L. (2003). Executive dysfunction and apathy predict functional impairment in Alzheimer disease. The American Journal of Geriatric Psychiatry, 11(2), 214–221. 10.1097/00019442-200303000-00012. 12611751

[ref11] Brown, T. (2014). Confirmatory factor analysis for applied research (2nd ed.). New York: Guilford Press.

[ref12] Brugulat-Serrat, A., Salvadó, G., Operto, G., Cacciaglia, R., Sudre, C. H., Grau-Rivera, O., et al. (2020). White matter hyperintensities mediate gray matter volume and processing speed relationship in cognitively unimpaired participants. Human Brain Mapping, 41(5), 1309–1322. 31778002 10.1002/hbm.24877 PMC7267988

[ref13] Burgmans, S., Gronenschild, E. H., Fandakova, Y., Shing, Y. L., Van Boxtel, M. P., Vuurman, E. F., et al. (2011). Age differences in speed of processing are partially mediated by differences in axonal integrity. NeuroImage, 55(3), 1287–1297. 21232618 10.1016/j.neuroimage.2011.01.002 PMC3057324

[ref14] Calamia, M., Roye, S., & Lemke, A. (2018). Does prior administration of the RBANS influence performance on subsequent neuropsychological testing? Applied Neuropsychology: Adult, 25(4), 340–343. 28323440 10.1080/23279095.2017.1299736

[ref15] Conners, K. C. (2004). Conner’s Continuous Performance Test (CPT II). Version 5 for windows. Technical Guide and Software Manual. North Tonawada, NY: Multi-Health Systems.

[ref17] Craik, F. I. (1986). A functional account of age differences in memory. Human Memory and Cognitive Capabilities: Mechanisms and Performances, 5, 409–422.

[ref18] Cullum, C. M., Saine, K., Chan, L. D., Martin-Cook, K., Gray, K. F., & Weiner, M. F. (2001). Performance-based instrument to assess functional capacity in dementia: The Texas Functional Living Scale. Cognitive and Behavioral Neurology, 14(2), 103–108. 11417663

[ref19] Cullum, C. M., Weiner, M. F., & Saine, K. C. (2009). Texas Functional Living Scale examiner’s manual. San Antonio, TX: Pearson.

[ref20] Delis, D., Kaplan, E., Kramer, J., & Ober, B. (2000). California Verbal Learning Test–second edition. San Antonio, TX: The Psychological Corporation.

[ref21] Delisle, J., & Braun, C. M. (2011). A context for normalizing impulsiveness at work for adults with attention deficit/hyperactivity disorder (combined type). Archives of Clinical Neuropsychology, 26(7), 602–613. 21653627 10.1093/arclin/acr043

[ref22] Drozdick, L. W., & Cullum, C. M. (2011). Expanding the ecological validity of the WAIS-IV and WMS-IV with the Texas Functional Living Scale. Assessment, 18(2), 141–155. 10.1177/1073191110382843. 20921288 PMC4389280

[ref24] Edwards, J. D., Bart, E., O’Connor, M. L., & Cissell, G. (2010). Ten years down the road: Predictors of driving cessation. The Gerontologist, 50(3), 393–399. 19726733 10.1093/geront/gnp127 PMC2867493

[ref25] Edwards, J. D., Ross, L. A., Wadley, V. G., Clay, O. J., Crowe, M., Roenker, D. L., et al. (2006). The useful field of view test: Normative data for older adults. Archives of Clinical Neuropsychology, 21(4), 275–286. 16704918 10.1016/j.acn.2006.03.001

[ref26] Farias, S. T., Harrell, C. N., Neumann, C., & Houtz, A. (2003). The relationship between neuropsychological performance and daily functioning individuals with Alzheimer’s disease: Ecological validity of neuropsychological tests. Archives of Clinical Neuropsychology, 18(6), 655–672. 10.1016/S0887-6177(02)00159-2. 14591439

[ref27] Glisky, E. L. (2007). Changes in Cognitive Function in Human Aging. In Riddle, D. R. (Ed.), Brain aging: Models, methods, and mechanisms (pp. 3–20). Boca Raton (FL): CRC Press/Taylor & Francis. 10.1201/9781420005523-1. 21204355

[ref28] Gold, D. A. (2012). An examination of instrumental activities of daily living assessment in older adults and mild cognitive impairment. Journal of Clinical and Experimental Neuropsychology, 34(1), 11–34. 22053873 10.1080/13803395.2011.614598

[ref29] Golden, C. J. (1978). Stroop Color And Word Test: A manual for clinical and experimental uses. Chicago, IL: Stoelting Co.

[ref30] Gonzalez, D. A., Soble, J. R., Marceaux, J. C., & McCoy, K. J. M. (2017). An evaluation of the Texas Functional Living Scale’s latent structure and subscales. Archives of Clinical Neuropsychology, 32(1), 104–109. 10.1093/arclin/acw082. 28122769

[ref31] Hall, J. R., Vo, H. T., Johnson, L. A., Barber, R. C., & O’Bryant, S. E. (2011). The link between cognitive measures and ADLs and IADL functioning in mild Alzheimer’s: What has gender got to do with it? International Journal of Alzheimer’s Disease, 2011, 276734. 10.4061/2011/276734. PMC3109554 21660245

[ref32] Hasher, L., Zacks, R. T., & May, C. P. (1999). Inhibitory control, circadian arousal, and age. In Gopher, D., & Zack, R. T. (Eds.), Attention and performance XVII (Vol. 17, pp. 653–675). Cambridge (MA): MIT Press. 10.7551/mitpress/1480.003.0032.

[ref33] Hedden, T., & Gabrieli, J. D. (2004). Insights into the ageing mind: A view from cognitive neuroscience. Nature Reviews Neuroscience, 5(2), 87–96. 14735112 10.1038/nrn1323

[ref34] Hinkin, C. H., Castellon, S. A., Durvasula, R. S., Hardy, D. J., Lam, M. N., Mason, K. I., et al. (2002). Mediation adherence among HIV+ adults. Neurology, 59, 1944–1950. 12499488 10.1212/01.wnl.0000038347.48137.67 PMC2871670

[ref35] Homack, S., & Riccio, C. A. (2006). Conners’ continuous performance test (2^nd^ ed.; CCPT-II). Journal of Attention Disorders, 9(3), 556–558. 16481673 10.1177/1087054705283578

[ref36] Hong, Z., Ng, K. K., Sim, S. K., Ngeow, M. Y., Zheng, H., Lo, J. C., et al. (2015). Differential age-dependent associations of gray matter volume and white matter integrity with processing speed in healthy older adults. NeuroImage, 123, 42–50. 26302672 10.1016/j.neuroimage.2015.08.034

[ref37] Ishikawa, H., Meguro, K., Ishii, H., Tanaka, N., & Yamaguchi, S. (2012). Silent infarction or white matter hyperintensity and impaired attention task scores in a nondemented population: The Osaki-Tajiri project. Journal of Stroke and Cerebrovascular Diseases, 21(4), 275–282. 20971655 10.1016/j.jstrokecerebrovasdis.2010.08.008

[ref38] Jefferson, A. L., Paul, R. H., Ozonoff, A., & Cohen, R. A. (2006). Evaluating elements of executive functioning as predictors of instrumental activities of daily living (IADLs). Archives of Clinical Neuropsychology, 21(4), 311–320. 10.1016/j.acn.2006.03.007. 16814980 PMC2746400

[ref39] Karr, J. E., Hofer, S. M., Iverson, G. L., & Garcia-Barrera, M. A. (2019). Examining the latent structure of the Delis–Kaplan executive function system. Archives of Clinical Neuropsychology, 34(3), 381–394. 29733343 10.1093/arclin/acy043

[ref40] Kerchner, G. A., Racine, C. A., Hale, S., Wilheim, R., Laluz, V., Miller, B. L., et al. (2012). Cognitive processing speed in older adults: Relationship with white matter integrity. PLoS One, 7(11), e50425. 10.1371/journal.pone.0050425. 23185621 PMC3503892

[ref41] Lassen-Greene, C. L., Steward, K., Okonkwo, O., Porter, E., Crowe, M., Vance, D. E., et al. (2017). Mild cognitive impairment and changes in everyday function over time: The importance of evaluating both speed and accuracy. Journal of Geriatric Psychiatry and Neurology, 30(4), 220–227. 28639877 10.1177/0891988717711807 PMC5812285

[ref42] Lowe, D. A., Nguyen, C. M., Copeland, C. T., & Linck, J. F. (2020). Factor analysis of the Texas Functional Living Scale in an outpatient clinical sample. Archives of Clinical Neuropsychology, 35(1), 116–121. 10.1093/arclin/acz005 30796805

[ref43] Lu, H., Chan, S. S., Fung, A. W., & Lam, L. C. (2016). Efficiency of attentional components in elderly with mild neurocognitive disorders shown by the attention network test. Dementia and Geriatric Cognitive Disorders, 41(1–2), 93–98. 26741695 10.1159/000441350

[ref44] Lu, H., Chan, S. S., & Lam, L. C. (2017). ‘Two-level’ measurements of processing speed as cognitive markers in the differential diagnosis of DSM-5 mild neurocognitive disorders (NCD). Scientific Reports, 7(1), 1–8. 28364127 10.1038/s41598-017-00624-8 PMC5428878

[ref45a] Lucas, J. A., Ivnik, R. J., Smith, G. E., Ferman, T. J., Willis, F. B., Petersen, R. C., & Graff-Radford, N. R. (2005). Mayo’s older African Americans normative studies: norms for Boston naming test, controlled oral word association, category fluency, animal naming, token test, wrat-3 reading, trail making test, Stroop test, and judgment of line orientation. The Clinical Neuropsychologist, 19(2), 243–269. 16019707 10.1080/13854040590945337

[ref45] Lustig, C., Hasher, L., & Tonev, S. T. (2006). Distraction as a determinant of processing speed. Psychonomic Bulletin & Review, 13(4), 619–625. 17201361 10.3758/bf03193972 PMC1764614

[ref46] MacPherson, S. E., Cox, S. R., Dickie, D. A., Karama, S., Starr, J. M., Evans, A. C., et al. (2017). Processing speed and the relationship between Trail Making Test-B performance, cortical thinning and white matter microstructure in older adults. Cortex, 95, 92–103. 28865241 10.1016/j.cortex.2017.07.021 PMC5637162

[ref47] Malloy-Diniz, L., Fuentes, D., Leite, W. B., Correa, H., & Bechara, A. (2007). Impulsive behavior in adults with attention deficit/hyperactivity disorder: Characterization of attentional, motor and cognitive impulsiveness. Journal of the International Neuropsychological Society, 13(4), 693–698. 17521490 10.1017/S1355617707070889

[ref48] Marson, D. (2015). Investigating functional impairment in preclinical Alzheimer’s disease. The Journal of Prevention of Alzheimer's Disease, 2(1), 4–6. 10.14283/jpad.2015.44 PMC4743883 26855935

[ref49a] McKay, C., Casey, J. E., Wertheimer, J., & Fichtenberg, N. L. (2007). Reliability and validity of the RBANS in a traumatic brain injured sample. Archives of Clinical Neuropsychology, 22, 91–98. 10.1016/j.acn.2006.11.003. 17141467

[ref49] Miyake, A., Friedman, N. P., Emerson, M. J., Witzki, A. H., Howerter, A., & Wager, T. D. (2000). The unity and diversity of executive functions and their contributions to complex “frontal lobe” tasks: A latent variable analysis. Cognitive Psychology, 41(1), 49–100. 10945922 10.1006/cogp.1999.0734

[ref50] Mlinac, M. E., & Feng, M. C. (2016). Assessment of activities of daily living, self-care, and independence. Archives of Clinical Neuropsychology, 31, 506–516. 27475282 10.1093/arclin/acw049

[ref51] Mortamais, M., Ash, J. A., Harrison, J., Kaye, J., Kramer, J., Randolph, C., et al. (2017). Detecting cognitive changes in preclinical Alzheimer's disease: A review of its feasibility. Alzheimer's & Dementia, 13(4), 468–492. 10.1016/j.jalz.2016.06.2365 27702618

[ref52] Muthén, L. K., & Muthén, B. O. (2012). Mplus User's guide: Statistical analysis with latent variables (7th ed.). Los Angeles, CA: Muthén & Muthén.

[ref53] Nguyen, C. M., Copeland, C. T., Lowe, D. A., Heyanka, D. J., & Linck, J. F. (2020). Contribution of executive functioning to instrumental activities of daily living in older adults. Applied Neuropsychology: Adult, 27(4), 326–333. 30646749 10.1080/23279095.2018.1550408

[ref54] Nickl-Jockschat, T., Kleiman, A., Schulz, J. B., Schneider, F., Laird, A. R., Fox, P. T., et al. (2012). Neuroanatomic changes and their association with cognitive decline in mild cognitive impairment: A meta-analysis. Brain Structure and Function, 217(1), 115–125. 21667303 10.1007/s00429-011-0333-x PMC4791066

[ref55] Nigg, J. T., Jester, J. M., Stavro, G. M., Ip, K. I., Puttler, L. I., & Zucker, R. A. (2017). Specificity of executive functioning and processing speed problems in common psychopathology. Neuropsychology, 31(4), 448. 28094999 10.1037/neu0000343 PMC5408314

[ref56] Nigg, J. T., Stavro, G., Ettenhofer, M., Hambrick, D. Z., Miller, T., & Henderson, J. M. (2005). Executive functions and ADHD in adults: Evidence for selective effects on ADHD symptom domains. Journal of Abnormal Psychology, 114(4), 706. 16351391 10.1037/0021-843X.114.3.706

[ref57] Nikolova, R., Demers, L., & Béland, F. (2009). Trajectories of cognitive decline and functional status in the frail older adults. Archives of Gerontology and Geriatrics, 48(1), 28–34. 10.1016/j.archger.2007.09.007. 17976840

[ref58] O’Brien, R. M. (2007). A caution regarding rules of thumb for variance inflation factors. Quality & Quantity, 41(5), 673–669. 10.1007/s11135-006-9018-6.

[ref59] Owsley, C., & McGwin, G., Jr. (2004). Association between visual attention and mobility in older adults. Journal of the American Geriatrics Society, 52(11), 1901–1906. 15507069 10.1111/j.1532-5415.2004.52516.x

[ref61] Randolph, C. (2012). RBANS update: Repeatable battery for the assessment of neuropsychological status. Bloomington, MN: NCS Pearson.

[ref62] Reitan, R. M. (1958). Validity of the Trail Making Test as an indicator of organic brain damage. Perceptual and Motor Skills, 8(3), 271–276. 10.2466/pms.1958.8.3.271.

[ref63] Rey-Mermet, A., & Gade, M. (2018). Inhibition in aging: What is preserved? What declines? A meta-analysis. Psychonomic Bulletin & Review, 25(5), 1695–1716. 29019064 10.3758/s13423-017-1384-7

[ref64] Roye, S., Castagna, P. J., Calamia, M., De Vito, A. N., Lee, T. H., & Greening, S. G. (2020). Relationships between multiple dimensions of executive functioning and resting-state networks in adults. Neuropsychologia, 141, 107418. 32169318 10.1016/j.neuropsychologia.2020.107418

[ref65] Salthouse, T. A. (1996). The processing-speed theory of adult age differences in cognition. Psychological Review, 103(3), 403. 8759042 10.1037/0033-295x.103.3.403

[ref66] Salthouse, T. A. (2003). Memory aging from 18 to 80. Alzheimer Disease & Associated Disorders, 17(3), 162–167. 14512830 10.1097/00002093-200307000-00008

[ref67] Salthouse, T. A. (2005). Relations between cognitive abilities and measures of executive functioning. Neuropsychology, 19(4), 532. 16060828 10.1037/0894-4105.19.4.532

[ref68] Salthouse, T. A. (2010). Major issues in cognitive aging. New York: Oxford University Press.

[ref69] Stawski, R. S., Sliwinski, M. J., & Hofer, S. M. (2013). Between-person and within-person associations among processing speed, attention switching, and working memory in younger and older adults. Experimental Aging Research, 39(2), 194–214. 23421639 10.1080/0361073X.2013.761556 PMC3622283

[ref70] Steiger, J. H. (1990). Structural model evaluation and modification: An interval estimation approach. Multivariate Behavioral Research, 25(2), 173–180. 26794479 10.1207/s15327906mbr2502_4

[ref71] Steinberg, B. A., Bieliauskas, L. A., Smith, G. E., & Ivnik, R. J. (2005). Mayo's older Americans normative studies: Age-and IQ-adjusted norms for the trail-making test, the stroop test, and MAE controlled oral word association test. The Clinical Neuropsychologist, 19(3–4), 329–377. 10.1080/13854040590945210. 16120535

[ref72] Strauss, E., Sherman, E. M., & Spreen, O. (2006). A compendium of neuropsychological tests: Administration, norms, and commentary. New York: Oxford University Press.

[ref73] Tombaugh, T. N. (1996). The Test Of Memory Malingering (TOMM): Normative data from cognitively intact and cognitively impaired individuals. Psychological Assessment, 9, 260–268. 10.1037/1040-3590.9.3.260.

[ref74] Turken, U., Whitfield-Gabrieli, S., Bammer, R., Baldo, J. V., Dronkers, N. F., & Gabrieli, J. D. (2008). Cognitive processing speed and the structure of white matter pathways: Convergent evidence from normal variation and lesion studies. NeuroImage, 42(2), 1032–1044. 18602840 10.1016/j.neuroimage.2008.03.057 PMC2630965

[ref75] Verbrugge, L. M., & Jette, A. M. (1994). The disablement process. Social Science & Medicine, 38(1), 1–14. 8146699 10.1016/0277-9536(94)90294-1

[ref76] Verhaeghen, P., & De Meersman, L. (1998). Aging and the Stroop effect: A meta-analysis. Psychology and Aging, 13(1), 120. 9533194 10.1037//0882-7974.13.1.120

[ref77] Wadley, V. G., Bull, T. P., Zhang, Y., Barba, C., Bryan, R. N., Crowe, M., ... & Kennedy, R. E. (2021). Cognitive Processing Speed Is Strongly Related to Driving Skills, Financial Abilities, and Other Instrumental Activities of Daily Living in Persons With Mild Cognitive Impairment and Mild Dementia. The Journals of Gerontology: Series A, 76(10), 1829–1838. 10.1093/gerona/glaa312. PMC8522472 33313639

[ref78] Wechsler, D. (2008). Wechsler Adult Intelligence Scale—Fourth edition. San Antonio, TX: Pearson Assessment.

[ref79] Weiner, M., Fields, J., Hynan, L., & Cullum, C. M. (2008). Annualized functional change in Alzheimer's disease participants and normal controls. The Clinical Neuropsychologist, 22(5), 801–806. 18609317 10.1080/13854040701750875 PMC2566778

[ref80] Weiner, M. F., Davis, B., Martin-Cook, K., Hynan, L. S., Saine, K. C., & Munro Cullum, C. (2007). A direct functional measure to help ascertain optimal level of residential care. American Journal of Alzheimer's Disease & Other Dementias, 22(5), 355–359. 10.1177/1533317507305174 PMC10846229 17959870

[ref81] Wolf, D., Zschutschke, L., Scheurich, A., Schmitz, F., Lieb, K., Tüscher, O., et al. (2014). Age-related increases in stroop interference: Delineation of general slowing based on behavioral and white matter analyses. Human Brain Mapping, 35(5), 2448–2458. 24038539 10.1002/hbm.22340 PMC6869565

